# Lactitol may improve the prognosis of hepatocellular carcinoma through the proliferation of Megasphaera as well as Bifidobacterium

**DOI:** 10.3389/fmed.2025.1567849

**Published:** 2025-05-14

**Authors:** Seigo Abiru, Yuki Kugiyama, Tomoyuki Suehiro, Yasuhide Motoyoshi, Akira Saeki, Shinya Nagaoka, Kazumi Yamasaki, Atsumasa Komori, Hiroshi Yatsuhashi

**Affiliations:** ^1^Department of Internal Medicine, NHO Saga Hospital, Saga, Japan; ^2^Clinical Research Center, NHO Nagasaki Medical Center, Nagasaki, Japan

**Keywords:** HCC, prognosis, prebiotics, nonabosorbable disaccharides, gut microbiota

## Abstract

**Background:**

Increasing evidence suggests that gut microbiota and their metabolites can modulate antitumor immunity. However, sufficient evidence from human studies is lacking. We evaluated the association of lactitol and lactulose as prebiotics with the progression of hepatocellular carcinoma (HCC).

**Methods:**

In Study 1, the effects of lactitol and lactulose on overall survival (OS) of patients with HCC with Child-Pugh scores of B or C were investigated in patients diagnosed at the Nagasaki Medical Center between 2003 and 2020. In Study 2, the effects of these substances on the gut microbiota of patients with cirrhosis were analyzed. Study 3 examined the effect of these substances on serum albumin levels in patients with cirrhosis.

**Results:**

In Study 1, a total of 321 patients were evaluated, and 55 pairs of Lactitol and Non-Lactitol groups and 80 pairs of Lactulose and Non-Lactulose groups were created using one to one propensity score matching. The Lactitol group showed a significant improvement (*p* < 0.05) in OS compared to the Non-Lactitol group, but the Lactulose group did not show any significance compared to the Non-Lactulose group. In Study 2, the number of Bifidobacterium was higher in the Lactitol group and the Lactulose group than in the Control group, but the number of Megasphaera was significantly higher only in the Lactitol group. In addition, in a study of 10 cases in which the gut microbiota was examined before and after lactitol use, an increase in Bifidobacterium and Megasphaera was observed after lactitol use. Study 3 found that lactitol had no beneficial effect on serum albumin levels.

**Conclusion:**

Lactitol may improve the prognosis of HCC through the proliferation of Megasphaera as well as Bifidobacterium.

## Introduction

The majority of hepatocellular carcinoma (HCC) occurs in patients with chronic liver disease (1). Therefore, the prognosis of HCC is influenced by many factors (2), including the degree of HCC progression at the time of diagnosis, ALT levels (3), degree of liver reserve function, initial treatment of HCC, and treatment of underlying liver disease (4) and treatment of recurrent HCC (5). For this reason, in many studies, multivariate analysis has been used to evaluate the efficacy of the initial treatment for HCC. In multivariate analysis, it is common to adjust for factors up to the time of enrollment, and the common method for evaluating the effectiveness of initial treatment is to adjust for factors up to the time of enrollment using propensity score matching and to compare overall survival (OS) and progression-free survival (PFS) after enrollment. However, in actual clinical practice, different treatments are also administered after the initial treatment, and the prognosis may differ greatly depending on the subsequent treatment. Therefore, prior to this study, we conducted a Cox proportional hazards regression analysis that included treatment factors after enrollment, and attempted to evaluate the impact of treatment factors on the prognosis of HCC and discover new hypotheses. In other words, we conducted a multivariate analysis that included factors such as procedures performed after enrollment [surgery, radiofrequency ablation (RFA), transcatheter arterial chemoembolization (TACE)], and the presence or absence of drugs mainly used for liver cirrhosis. The results varied depending on the factors entered and the number of cases, but procedures such as surgery and RFA and the use of antiviral drugs were always extracted as independent factors, and nonabsorbable disaccharides (NADs), particularly lactitol, were often extracted as independent factors. NADs have been used since 1996 for the treatment of hepatic encephalopathy in patients with decompensated cirrhosis. They exert their therapeutic effect by affecting the gut microbiota and we considered the possibility that the gut microbiota induced by lactitol might have a beneficial effect.

The gut microbiota is recognized as an important new player in the pathogenesis of intestinal and extraintestinal diseases (6, 7). Several liver diseases such as alcoholic liver disease, non-alcoholic liver disease, primary sclerosing cholangitis are associated with gut dysbiosis (8, 9). Accumulating evidence suggests a key role of the gut microbiota in promoting the progression of liver disease and the development of HCC (10, 11). It has also been shown that probiotic-modulated gut microbiota suppresses HCC growth in mice (12). NADs have been classified as prebiotics and can beneficially affect microbiota composition. Lactitol is the second-generation NAD (13), and is similar to lactulose in that it promotes the growth of bifidobacteria and lactobacilli. However, previous studies revealed that lactitol increases less small number strains of intestinal bacteria compared to lactulose (14, 15). Increasing evidence suggests that gut microbiota and their metabolites can modulate not only antitumor immunity (16–18), but also the response to cancer therapy and susceptibility to toxic side effects (19). Thus, probiotics, prebiotics and synbiotics are expected to be useful for the prevention and treatment of gastrointestinal cancer, biliary tract cancer, pancreatic cancer and the like, including HCC (20–22). However, sufficient evidence from human studies is lacking (23).

To date, NADs have been used in hepatic encephalopathy. Therefore, in this study, the effect of NADs on the prognosis of HCC patients with decompensated liver cirrhosis was evaluated using propensity score matching. In addition, to explore the causes of the results, the effects of lactitol and lactulose on the gut microbiota of patients with liver cirrhosis were investigated using next-generation sequencing analysis.

## Patients and methods

### Study design

This study consisted of three parts, Study 1, Study 2, and Study 3 which were conducted at the Nagasaki Medical Center. Study 1 was a retrospective observational study that investigated the effects of lactitol and lactulose on overall survival (OS) in patients with HCC who had developed decompensated liver cirrhosis. Study 2 was a prospective study that investigated the effects of lactitol and lactulose on the gut microbiota of patients with liver cirrhosis. Study 3 was a retrospective observational study that investigated the effects of lactitol and lactulose on liver reserve function in patients with cirrhosis but no HCC.

### Patients

Study 1 was conducted from January 2003 to December 2020 and included patients with Child B or C liver cirrhosis who developed HCC and survived more than 30 days. The study evaluated the effect of taking NADs after the diagnosis of HCC on OS. Patients who took lactitol or lactulose for more than 30 days were included in “Lactitol group” or “Lactulose group,” respectively, and patients who took them for more than 30 days at different times were included in “Both group,” and patients who took neither formulation for less than 30 days were included in the “Non group,” and OS was examined. In addition, the effect of taking lactitol or lactulose on OS was examined using propensity score matching.

Study 2 involved the analysis of the gut microbiota of patients with liver cirrhosis. From April 2017 to March 2021, patients who provided written consent to participate in the study at the Nagasaki Medical Center had their feces stored in a bag with ice for a few hours, and then stored in a freezer at −80°C. The samples were then sent frozen to Technosuruga Laboratory (Shizuoka City, Japan) for analysis of the gut microbiota. The analysis of the gut microbiota was carried out using the following methods. DNA extraction was carried out according to the method reported previously (24). The V3-V4 region of the 16S rDNA of bacteria and archaea was amplified using the Pro341F/Pro805R primer and the dual index method (24, 25). The barcoded amplicons were sequenced in paired-end 2 × 284 bp reads using the MiSeq system and the MiSeq Reagent Kit version 3 (600 cycle) chemistry. The paired-end sequencing reads were merged using the default settings of the fastq-join program (26). Using the FASTX toolkit, only the merged reads with a quality score of 20 or higher were extracted for more than 99% of the sequences (27). Chimeric sequences were removed using usearch61 (28, 29). For classification, we used the Ribosomal Database Project (RDP) Classifier ver 2.11 (30) and the Technosuruga Laboratory Microbial Identification Database (DB-BA) ver 13.0 (Technosuruga Laboratory, Japan) (31). For the RDP Classifier and the DB-BA database, respectively, were used to identify sequences with a confidence level of ≥0.8 and a homology of ≥97%, using the Metagenome@KIN ver 2.2.1 analysis software (World Fusion, Japan). Among these, sequences with a homology search result of less than 97% against the microbial identification database were defined as “rejected hit,” and sequences with a homology rate of 97% or more against the microbial identification database, but for which the taxonomic group could not be determined because they were hits with the same homology rate, were defined as “not determined.” Study 2a was conducted on patients diagnosed with liver cirrhosis and with an albumin (Alb) level of less than 3.5 g/dL. Patients who took lactitol or lactulose for at least 5 days were included in the Lactitol group or the Lactulose group, respectively, and patients who did not take the NAD were included in the Control group. Study 2b analyzed the changes in the gut microbiota of 10 patients of liver cirrhosis who took lactitol before and after taking lactitol.

Study 3 examined the change in serum Alb levels before and after lactitol and lactulose intake in patients with cirrhosis who had not developed HCC between 2003 and 2020 and who had taken NADs for at least 6 months, and in whom serum Alb levels were measured 6 months before and 6 months after taking NADs.

### Data collection

Data from blood tests, biochemical tests, liver resection, RFA, TACE, and other procedures, as well as prescription drugs, were collected from the Data Ware House (DWH) of the Nagasaki Medical Center’s electronic medical records and organized using the pivot function in Excel.

### Statistical analysis

For Study 1, differences in baseline characteristics between the groups were evaluated using the chi-square test for categorical variables and the Mann–Whitney U test for continuous variables. Kaplan–Meier analysis was performed for the Lactitol group, Lactulose group, Both groups, and the Non group. Propensity score matching was used to compare OS between the Lactitol group and the non-Lactitol group, and between the Lactulose group and the non-Lactulose group. One-to-one propensity score matching was performed to adjust for measured confounding factors, regardless of significance. Logistic regression models were used to predict the probability that each patient would be given lactitol. The following variables were used as predictors: age, sex, virology (hepatitis B virus [HBV], hepatitis C virus [HCV], HBV + HCV, non-B non-C hepatitis [nBnC]), Child-Pugh score, BCLC stage, total bilirubin, aspartate aminotransferase (AST), alanine aminotransferase (ALT), albumin, prothrombin, platelet count, alpha-fetoprotein (AFP), des-gamma-carboxyprothrombin (DCP), interferon (IFN) therapy, direct-acting antiviral (DAA) therapy, RFA treatment, liver resection, TACE, and the use of multi-kinase inhibitors were examined as items. Each patient who received lactitol was matched with a patient who did not receive lactitol using a 25% caliper of the standard deviation of the propensity score on the logit scale. Similarly, each patient who received lactulose was matched in the same way. OS was defined as the time from the date of HCC diagnosis to the date of death or last follow-up. Survival analysis was performed using the Kaplan–Meier method, and differences were tested using the log-rank test. For Study 2a, comparisons between Lactitol, Lactulose, and the Control group were made using the Mann–Whitney U test. For Study 2b and Study 3, the Wilcoxon signed rank test was used.

All analyses were performed using IBM SPSS version 25 and StatFlex version 7. A two-tailed *p* value of less than 0.05 was considered statistically significant.

### Ethical considerations

This study was conducted in accordance with the Declaration of Helsinki, and the research protocol was approved by the Ethics Committee of the Nagasaki Medical Center, National Hospital Organization (Approval No. 30141). Consent to participate in Study 1 and Study 3 was obtained using an opt-out approach, and written consent to participate in Study 2 obtained.

## Results

### Study 1

**Figure 1:**
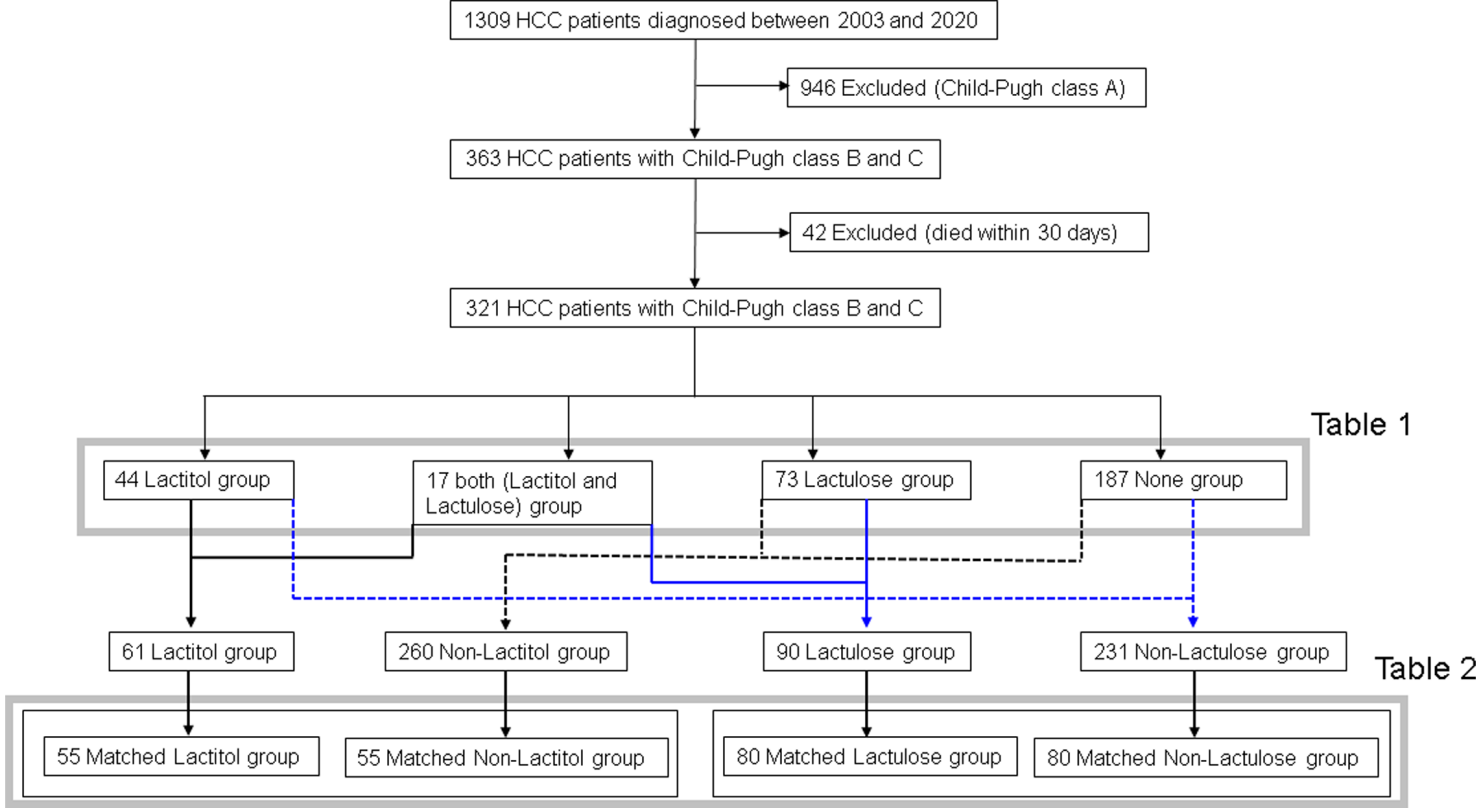
Flowchart showing participant recruitment in Study 1. HCC, hepatocellular carcinoma.

**Figure 2:**
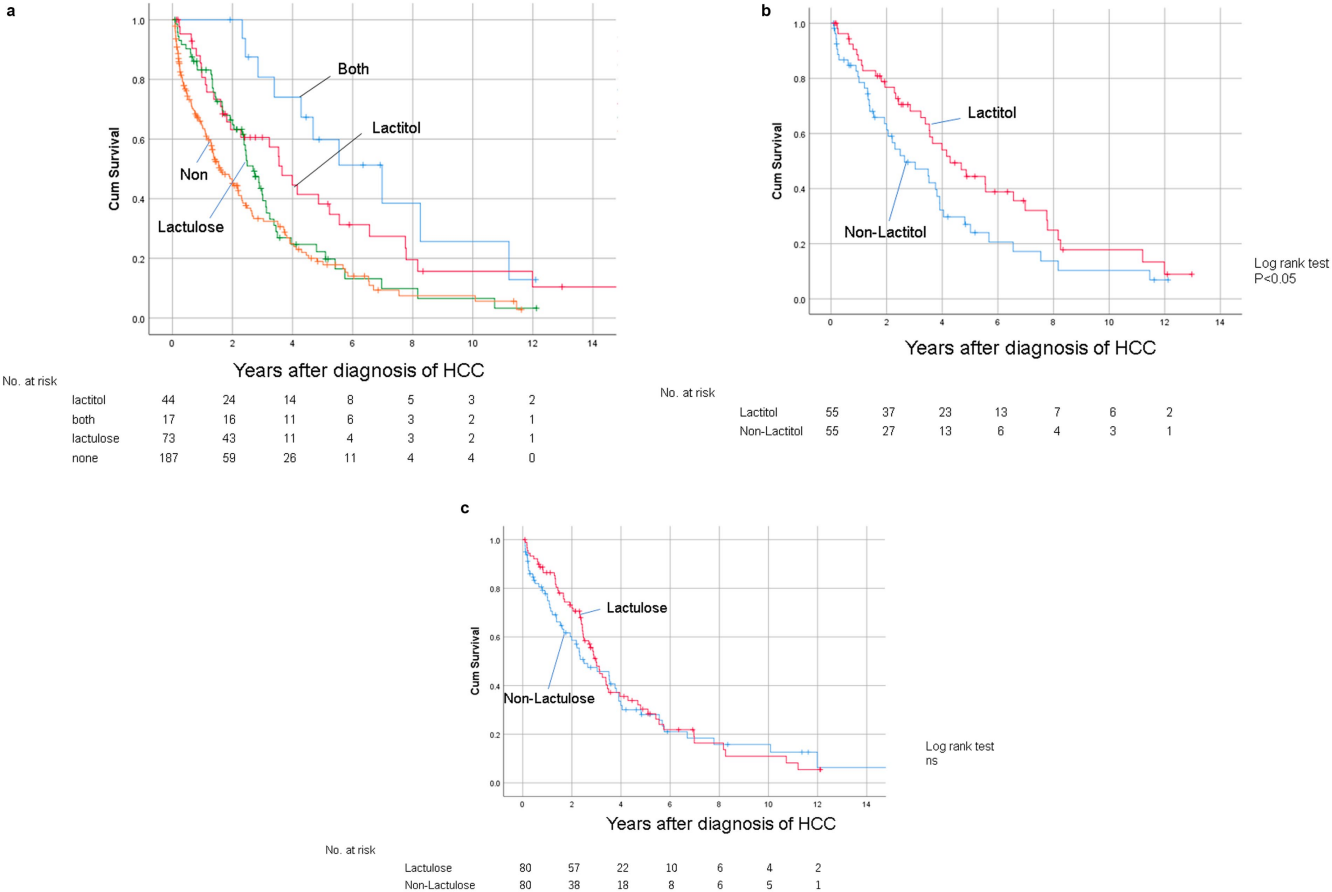
**(a)** Kaplan–Meier curves for overall survival after diagnosis of HCC in the unmatched data in Study 1. **(b)** Kaplan–Meier curve for OS after diagnosis of HCC between the Lactitol group and the Non Lactitol group in the matched data in Study 1. **(c)** Kaplan–Meier curve for OS after diagnosis of HCC between the Lactulose group and the Non Lactulose group in the matched data in Study 1.

Figure 1 shows the flowchart of participant recruitment. A total of 321 patients were confirmed to meet the inclusion criteria for Study 1. Of these, 13.7% (*n* = 44) were taking lactitol only (Lactitol group), 5.3% (*n* = 17) were taking lactitol and lactulose (Both group), 22.7% (*n* = 73) were taking lactulose only (Lactulose group), and 58.2% (*n* = 187) were not taking either lactitol or lactulose (Non group). The background of the patients is shown in Table 1. There were many factors that showed significant differences among the four groups, and the variation was particularly strong in the “Both” and “Non” groups. Figure 2a shows the Kaplan–Meier curves for each group. In the unadjusted analysis, the Both group showed a significant increase in OS compared to the Lactulose group and the Non group. The Lactitol group showed a significant increase in OS compared to the Non group, but there was no significant difference compared to the Lactulose group. In addition, as shown in Figure 1, one-to-one propensity score matching was used to create 55 pairs of Lactitol and Non Lactitol groups and 80 pairs of Lactulose and Non-Lactulose groups. Table 2 shows the background of the patients. The Lactitol group showed a significant improvement (*p* < 0.05) in OS compared to the Non-Lactitol group (Figure 2b). The Lactulose group did not show any significance compared to the Non-Lactulose group (Figure 2c).

**Table 1:** Summary of patient and treatment characteristics in Study 1.

Characteristics	Lactitol	Lactulose	Non	Both	*p*-value
	*n* = 44	*n* = 73	*n* = 187	*n* = 17	
Sex (male), n (%)	30 (70)	37 (51)	137 (73)	9 (53)	<0.005
Age at diagnosis (IQR)	70 (62.0–75.0)	62 (62.0–74.0)	66 (59.3–76.0)	60 (55.5–66.0)	ns
Virology					
B, *n* (%)	11 (25)	13 (18)	42 (22)	7 (41)	ns
C, *n* (%)	19 (43)	39 (53)	94 (50)	7 (41)	
nBnC, *n* (%)	14 (32)	21 (29)	47 (25)	2 (12)	
B + C, *n* (%)	0 (0)	0 (0)	4 (2)	1 (6)	
Child-Pugh score					
B, *n* (%)	42 (95)	58 (79)	164 (88)	14 (82)	ns
C, *n* (%)	2 (5)	15 (21)	23 (12)	3 (18)	
BCLC					
A, *n* (%)	30 (68)	46 (63)	84 (45)	14 (82)	<0.005
B, *n* (%)	10 (23)	19 (26)	58 (31)	2 (12)	
C, *n* (%)	4 (9)	8 (11)	45 (24)	1 (6)	
total bilirubin, mg/dL (IQR)	2.0 (1.2–2.5)	1.6 (1.2–2.4)	1.4 (0.9–2.2)	1.9 (1.5–2.0)	ns
AST, IU/ml (IQR)	54 (39–82)	59 (43–83)	64 (46–101)	53 (27–79)	ns
ALT, IU/ml (IQR)	38 (28–64)	35 (27–49)	41 (27–67)	24 (16–47)	<0.05
Albumin, g/dL (IQR)	3.2 (2.8–3.5)	3.0 (2.6–3.3)	3.2 (2.8–3.5)	3.1 (2.7–3.5)	ns
Prothrombin time, % (IQR)	65 (59–70)	62 (55–67)	69 (60–75)	57 (49–68)	<0.0005
Platelet counts, 10^4^/μL (IQR)	8.0 (5.6–11.3)	7.3 (5.7–12.5)	9.7 (6.8–14.8)	6.1 (5.5–13.7)	<0.05
AFP, ng/mL (IQR)	13 (5–10)	21 (9–80)	41 (12–792)	17 (7–44)	<0.005
DCP, mAU/L (IQR)	71 (25–411)	91 (28–414)	190 (30–2,874)	80 (31–471)	ns
Treatment					
IFN, *n* (%)	6 (12)	2 (3)	20 (11)	2 (11)	n.s.
DAA, *n* (%)	8 (18)	15 (20)	23 (12)	4 (24)	n.s.
RFA, *n* (%)	2 (5)	3 (4)	7 (4)	2 (12)	<0.05
liver resection, *n* (%)	15 (35)	21 (29)	31 (17)	6 (35)	n.s.
TACE, *n* (%)	34 (77)	50 (69)	111 (59)	8 (47)	n.s.
Radiation therapy, *n* (%)	9 (21)	3 (4)	10 (5)	2 (12)	<0.005
Multi-kinase inhibitors, *n* (%)	1 (2)	2 (3)	4 (2)	2 (12)	n.s.
Lactitol, day (IQR)	159 (74–376)	15 (12–19)	20 (12–21)	534 (102–934)	<0.00001
Lactulose, day (IQR)	7 (7–14)	166 (74–379)	17 (7–21)	208 (97–418)	<0.00001

**Table 2:** Summary of patient and treatment characteristics: propensity score matching in Study 1.

Characteristics	Lactitol	Non-lactitol	*p*-value	Lactulose	Non-lactulose	*p*-value
	*n* = 55	*n* = 55		*n* = 80	*n* = 80	
Sex (male), *n* (%)	35 (65)	43 (78)	ns	45 (56)	51 (64)	ns
Age at diagnosis (IQR)	66 (57.5–74.2)	66 (56.8–74.1)	ns	67 (60–73)	65 (56–73)	ns
Virology						
B, *n* (%)	15 (27)	26 (47)	<0.005	17 (21)	29 (36)	<0.05
C, *n* (%)	24 (44)	7 (13)		42 (53)	26 (33)	
nBnC, *n* (%)	22 (27)	22 (40)		20 (25)	25 (31)	
B + C, n (%)	1 (2)	0 (0)		1 (1)	0 (0)	
Child-Pugh score						
B, *n* (%)	50 (91)	50 (91)	ns	68 (85)	65 (81)	ns
C, *n* (%)	5 (9)	5 (9)		12 (15)	15 (19)	
BCLC						
A, *n* (%)	39 (71)	33 (60)	ns	52 (65)	47 (59)	ns
B, *n* (%)	11 (20)	16 (29)		19 (24)	24 (30)	
C, *n* (%)	5 (9)	6 (11)		9 (11)	9 (11)	
Total bilirubin, mg/dL (IQR)	1.8 (1.4–2.1)	1.6 (1.0–1.9)	ns	1.7 (1.3–2.3)	1.8 (1.0–2.4)	ns
AST, IU/ml (IQR)	54 (36–79)	53 (38–79)	ns	60 (42–82)	58 (39–82)	ns
ALT, IU/ml (IQR)	34 (24–55)	33 (25–50)	ns	35 (27–51)	38 (24–47)	ns
Albumin, g/dL (IQR)	3.2 (2.8–3.5)	3.2 (2.8–3.5)	ns	3.1 (2.8–3.4)	3.1 (2.8–3.5)	ns
Prothrombin time, % (IQR)	64 (54–69)	65 (60–72)	ns	62 (57–69)	63 (52–70)	ns
platelet counts, 10^4^/μL (IQR)	7.4 (5.5–10.6)	9.1 (6.1–12.2)	ns	6.1 (5.7–10.9)	7.6 (5.2–9.9)	ns
AFP, ng/mL (IQR)	12 (5–80)	19 (8–190)	ns	19 (9–60)	22 (7–127)	ns
DCP, mAU/L (IQR)	71 (27–353)	97 (21–611)	ns	78 (28–351)	56 (17–184)	ns
Treatment						
IFN, *n* (%)	11 (20)	4 (7)	ns	17 (21)	11 (14)	ns
DAA, *n* (%)	4 (7)	2 (4)	ns	4 (5)	2 (3)	ns
RFA, *n* (%)	20 (36)	19 (35)	ns	23 (29)	28 (35)	ns
liver resection, *n* (%)	7 (13)	7 (13)	ns	4 (5)	6 (7.5)	ns
TACE, *n* (%)	37 (67)	39 (71)	ns	54 (68)	54 (68)	ns
Radiation therapy, *n* (%)	6 (11)	3 (5)	ns	4 (5)	2 (3)	ns
Multi-kinase inhibitors, *n* (%)	2 (4)	2 (4)	ns	2 (3)	2 (3)	ns
NAD, day (IQR)	194 (77–582)	0	<0.00005	0 (0–4)	174 (73–381)	<0.00005
Both NADs, *n* (%)	17 (30)	16 (29)	ns	13 (16)	19 (24)	ns

### Study 2

**Figure 3:**
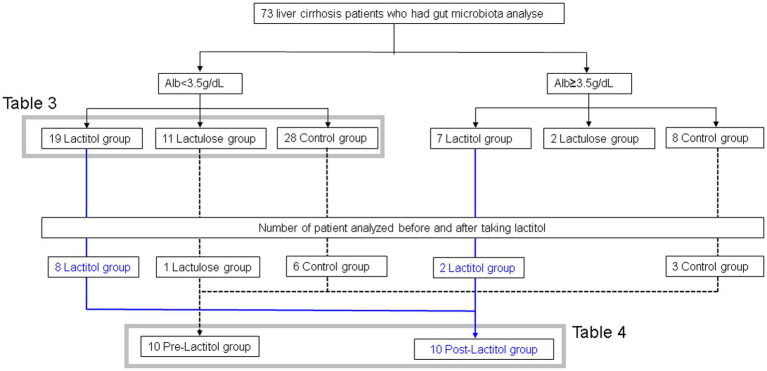
Flowchart showing participant recruitment in Study 2.

Figure 3 shows the flowchart of participant recruitment. We analyzed the gut microbiota of 73 patients with liver cirrhosis. Of these, we conducted Study 2a on 56 patients with liver cirrhosis with an Alb level of less than 3.5 g/dL. We conducted Study 2b on 10 patients with liver cirrhosis who had their gut microbiota analyzed before and after taking lactitol.

#### Study 2a

**Figure 4:**
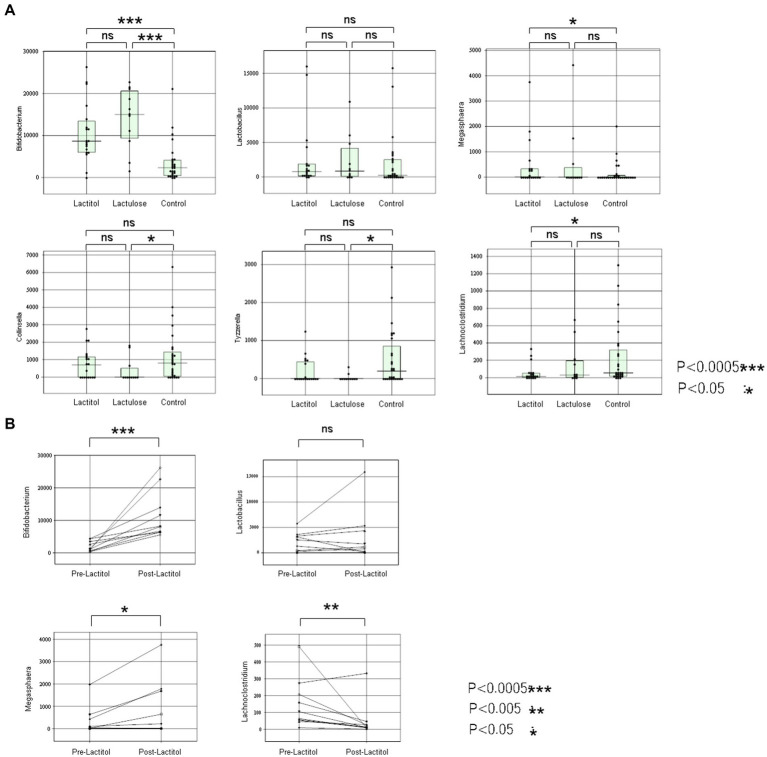
**(a)** Comparison of the lactitol, lactulose, and control groups using the Mann–Whitney test in Study 2a. **(b)** The changes in the gut microbiota of 10 patients who took lactitol before and after taking lactitol in Study 2b.

The patient backgrounds examined are shown in Table 3. There were no significant differences except that there were more HCC patients in the Lactitol group. The number of Bifidobacterium was significantly higher in the Lactitol and Lactulose groups. The number of Megasphaera was significantly higher in the Lactitol group. The number of Lachnoclostridium was significantly lower in the Lactitol group. In addition, the number of Collinsella and Tyzzerella were significantly lower in the Lactulose group. Among the 228 bacterial genera that could be examined, there were no significant differences in Lactobacillus or other bacteria in the Lactulose and Lactitol groups (Figure 4a).

**Table 3:** Patient characteristics in Study 2a.

Characteristics	Lactitol group	Lacutulose group	Control group	*p* value
	*n* = 19	*n* = 11	*n* = 28	
Sex (male), *n* (%)	15 (64)	6 (55)	19 (68)	ns
Age (IQR)	69.0 (64.0–73.0)	69.0 (66.31–73.5)	68.5 (57.0–72.5)	ns
Virology				
B, *n* (%)	1 (5)	0 (0)	4 (14)	ns
C, *n* (%)	8 (42)	3 (27)	3 (10)	<0.05
nBnC, *n* (%)	10 (53)	7 (64)	20 (71)	ns
HCC, *n* (%)	14 (74)	4 (36)	9 (32)	<0.05
Total bilirubin, mg/dL (IQR)	1.6 (0.8–2.9)	2.1 (1.4–2.9)	1.8 (0.7–2.9)	ns
AST, IU/ml (IQR)	40 (28.8–123.0)	40 (33.8–47.8)	48 (36.0–82.5)	ns
ALT, IU/ml (IQR)	28 (15.5–60.0)	26 (18.3–35.0)	30 (20.0–72.0)	ns
Albumin, g/dL (IQR)	3.1 (2.4–3.3)	2.5 (2.3–3.1)	2.9 (2.6–3.2)	ns
Prothrombin time, % (IQR)	68.9 (38.0–77.0)	47.5 (41.2–54.0)	63.0 (46.2–81.4)	ns
Platelet counts, 10^4^/μL (IQR)	11.2 (6.2–14.0)	8.7 (7.6–12.7)	12.2 (6.9–15.1)	ns

#### Study 2b

The background of the 10 patients for whom the gut microbiota analysis was performed before and after lactitol administration is shown in Table 4. The three bacteria that were significant factors in Study 2a showed significant increases or decreases after administration. In other words, the number of Bifidobacterium and Megasphaera showed a significant increase, while the number of Lachnoclostridium showed a significant decrease (Figure 4b).

**Table 4:** Patient characteristics in Study 2b.

Characteristics	Pre-Lactitol	Post-Lactitol	*p* value
	*n* = 10	*n* = 10	
Sex (male), *n* (%)	8 (80)	8 (80)	–
Age (IQR)	66 (56–77)	66 (56–77)	–
Virology			
B, *n* (%)	3 (30)	3 (30)	–
C, *n* (%)	2 (20)	2 (20)	
nBnC, *n* (%)	5 (50)	5 (50)	
HCC, *n* (%)	6 (60)	6 (60)	–
Total bilirubin, mg/dL (IQR)	0.7 (0.6–2.3)	0.7 (0.6–2.2)	ns
AST, IU/ml (IQR)	34 (25–46)	32 (25–51)	ns
ALT, IU/ml (IQR)	21 (17–32)	22 (15–56)	ns
Albumin, g/dL (IQR)	3.2 (3.1–4.0)	3.2 (2.5–3.4)	ns
Prothrombin time, % (IQR)	85 (48–92)	75 (56–83)	ns
Platelet counts, 10^4^/μL (IQR)	9.5 (7.0–16.4)	12.5 (9.1–16.5)	ns
Numbers of days of lactitol use (IQR)	–	64 (9–105)	–

### Study 3

**Figure 5:**
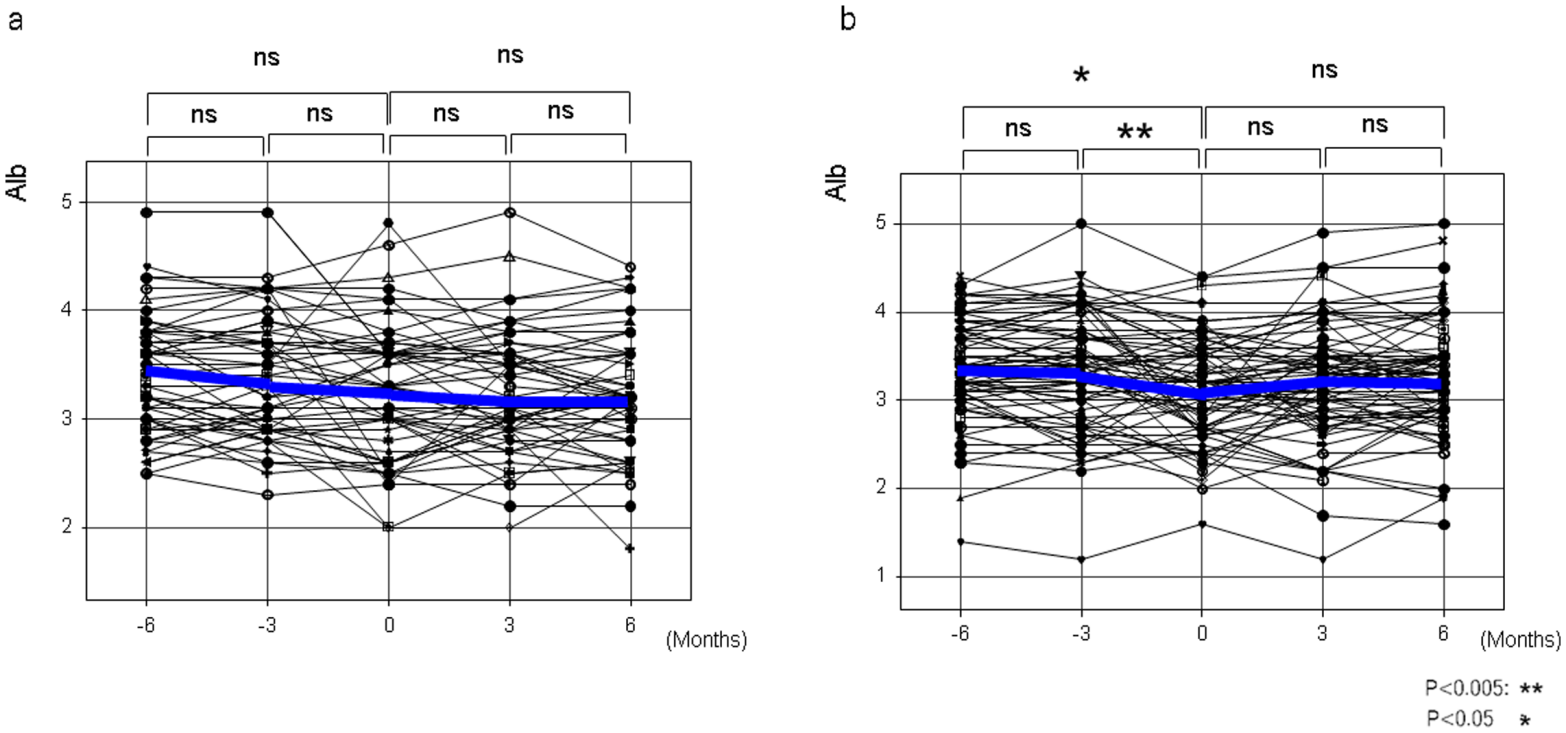
The changes in serum albumin levels before and after taking lactitol or lactulose in Study 3. **(a)** Lactitol intake. **(b)** Lactulose intake.

In patients with liver cirrhosis who had not developed HCC and who had taken NADs for at least 6 months, serum albumin levels were measured in 54 lactitol and 70 lactulose patients from 6 months before taking NADs to 6 months after taking NADs (Table 5). As shown in Figure 5, at least lactitol had no beneficial effect on serum albumin levels.

**Table 5:** Patient characteristics in Study 3.

Characteristics	Lactitol *n* = 54	Lactitulose *n* = 70
Sex (male), *n* (%)		
Virology		
B, *n* (%)	2 (4)	9 (13)
C, *n* (%)	19 (35)	29 (41)
B + C	0 (0)	3 (4)
nBnC, *n* (%)	29 (54)	23 (33)
AIH + PBC, *n* (%)	4 (7)	6 (9)

## Discussion

In this study, a propensity score matching analysis was conducted on HCC patients with decompensated liver cirrhosis, and while there was no significant increase in survival time for lactulose users, lactitol users showed a significant increase in survival time compared to non-lactitol users. In addition, in the analysis of gut microbiota in patients with cirrhosis, the number of Bifidobacterium and Megasphaera in the gut microbiota was found to be significantly higher in the lactitol group, and in the study before and after lactitol intake, it was found that lactitol administration significantly increased not only Bifidobacterium but also Megasphaeraga. In addition, lactitol was not found to have a beneficial effect on liver reserve function.

Previous observational studies have shown that statins are associated with reduced risk of various cancers (32, 33). However, it has been shown that these results might be explained by selection bias and immortal-time bias (34). In order to avoid such biases, we excluded HCC patients with a Child-Pugh class A score and who died within 30 days, since we defined patients treated for 30 days or more as treatment groups. Propensity score matching is used to emulate a randomized trial. Thus, it is thought that factors after enrollment should be excluded. However, the prognosis of HCC depends on treatments such as liver resection, RFA, and TACE. Therefore, we contained treatments factor after enrollment. The gut microbiota contributes to disease progression at various stages of liver disease and may promote the development of HCC throughout all these stages (10). In preliminary studies, lactitol and lactulose were often extracted as independent factors in COX proportional hazard regression analysis, and we expected that both lactitol and lactulose, which are prebiotics, would be correlated with the prognosis of HCC. Surprisingly, in the propensity score analysis, we found that only lactitol users had a longer survival time for HCC than non-lactitol users. Lactitol, similar to lactulose, can reduce the level of endotoxin in the plasma by improving the gut microbiota (35, 36). This may have a beneficial effect on liver reserve function in patients with cirrhosis, but Study 3 did not find a beneficial effect of lactitol on liver reserve function. It has been reported that the gut microbiota regulated by lactitol and lactulose are different (14, 15), but this has not been investigated using next-generation sequencing analysis in recent years, so we conducted Study 2. Analysis at the “species” level did not identify any significant bacteria. We considered that the reason for this was that, when examining at the “species” level, (1) the number of bacteria per species was small due to the detailed classification, and (2) there were a considerable number of bacteria that could not be classified, such as “rejected hit” and “not determined,” so we conducted an examination at the “genus” level. Even at the “genus” level, there were “rejected hit” and “not determined,” but in lactitol, in addition to Bifidobacterium, Megasphaera showed a significant increase, and Lachnoclostridium showed a significant decrease. On the other hand, in lactulose, Bifidobacterium was significantly higher, but there was no significant difference in Megasphaera and Lachnoclostridium. There are some reports on the anti-tumor effects of Bifidobacterium (37–39), such as its ability to enhance tumor immunity in cancer and its synergistic effect when used in combination with immune checkpoint inhibitors. In addition, it has recently been reported that Megasphaera is present in the tumors of patients with pancreatic ductal adenocarcinoma (PDAC) who have survived for a long time (40), and that short-chain fatty acids such as butyrate and pentanoate, which are induced by Megasphaera, regulate the response of CD8-positive T cells and enhance the effects of cancer immunotherapy (41). This suggests that lactitol may have improved the prognosis of HCC by increasing the growth of Megasphaera as well as Bifidobacterium. However, in the paper by Lu et al. (42), they conducted shotgun metagenomic analysis and reported that lactitol supplementation adjusted the gut microbiota of patients with cirrhosis, but there is no mention of Megasphaera in this paper. There are differences between races, and it is possible that this may affect the treatment effect. Lachnostridium, which showed a significant decrease in the lactitol group, and Collinsella and Tyzzerella, which were significantly lower in the lactulose group, have also been reported to have a beneficial effect on cancer (43, 44), and a decrease in these bacteria may be related to a decrease in anti-tumor effects. Therefore, the combination of lactitol and lactulose may enhance the anti-tumor effect. Study 1 included cases where effective treatments such as surgery, RFA, and multi-kinase inhibitors were not possible due to decreased liver reserve function, and in the future, it is hoped that studies will be conducted on the combination of multi-kinase inhibitors or immune checkpoint inhibitors and lactitol in HCC cases where liver reserve function is maintained. In addition, studies on the combination of chemotherapy and lactitol are also desired in cases of digestive organ cancers such as pancreatic cancer.

## Limitation

This study uses propensity score matching, but differs from the usual method in that it includes analysis of treatment after enrollment. Therefore, this study is at the level of hypothesis discovery. To verify the hypothesis, that is, to prove the effect of lactitol, a prospective study is needed. In addition, the gut microbiota analysis performed in Study 2 cannot be said to have sufficiently extracted bacteria useful for HCC due to the following factors: (1) the cases analyzed in Study 1 were different, (2) patients with cirrhosis without HCC were also included, and (3) the number of cases was small. The gut microbiota shows complex relationships, and further investigation is needed.

## Conclusion

Lactitol may improve the prognosis of HCC through the proliferation of Megasphaera as well as Bifidobacterium. This is expected to have an effect not only on HCC, but also on digestive cancers including pancreatic cancer.

## Data Availability

The original contributions presented in the study are included in the article material, further inquiries can be directed to the corresponding author.
